# Epidemiology and Genetic Characterization of Noroviruses among Adults in an Endemic Setting, Peruvian Amazon Basin, 2004–2011

**DOI:** 10.1371/journal.pone.0131646

**Published:** 2015-07-10

**Authors:** Sarah-Blythe Ballard, Erik J. Reaves, C. Giannina Luna, Maria E. Silva, Claudio Rocha, Kristen Heitzinger, Mayuko Saito, Sonia Apaza, Susan Espetia, David L. Blazes, Drake H. Tilley, Rene C. Guzmán Aguilar, Robert H. Gilman, Daniel G. Bausch

**Affiliations:** 1 Department of International Health, Johns Hopkins Bloomberg School of Public Health, Baltimore, Maryland, United States of America; 2 Emerging Infectious Diseases Department, United States Medical Research Unit No. 6, Lima, Peru; 3 Bacteriology Department, United States Medical Research Unit No. 6, Lima, Peru; 4 Department of Epidemiology, University of Washington, Seattle, Washington, United States of America; 5 Department of Virology, Tohoku University Graduate School of Medicine, Sendai, Japan; 6 Laboratorios de Investigación y Desarrollamiento, Universidad Peruana Cayetano Heredia, Lima, Peru; 7 Department of Preventive Medicine and Biometrics, Uniformed Services University of the Health Sciences, Bethesda, Maryland, United States of America; 8 Policlínico Militar de Chorrillos, Chorrillos, Peru; 9 Asociación Benéfica PRISMA, Lima, Peru; 10 Department of Tropical Medicine, Tulane School of Public Health and Tropical Medicine, New Orleans, Louisiana, United States of America; University of Parma, ITALY

## Abstract

**Background:**

Successful vaccination strategies against norovirus will require understanding the burden of disease and relevant genotypes in populations. However, few data are available from cohort studies of adults living in low- and middle-income countries (LMIC).

**Materials and Methods:**

We conducted a nested case-control study within a Peruvian military cohort to characterize the burden of norovirus infection, predominant genotypes, and associated symptoms from 2004 through 2011. Randomly selected case and control stools were tested for norovirus, bacteria, and parasites. The odds ratio of the association between norovirus infection and diarrhea was estimated using multiple logistic regression and co-infection adjusted attributable fractions were calculated.

**Results:**

Of the 3,818 cohort study participants, 624 developed diarrhea. Overall and norovirus-associated diarrhea incidence rates were 42.3 and 6.0 per 100 person-years, respectively. The most prevalent norovirus genogroup was GII (72.5%, 29/40), which was associated with diarrhea (AOR 3.4, 95% CI: 1.3–8.7, *P* = 0.012). The co-infection adjusted GII attributable fraction was 6.4%.

**Discussion:**

Norovirus was a frequent cause of diarrhea in an adult population followed longitudinally in an LMIC setting. Vaccine strategies should consider targeting adults in endemic settings and special populations that could serve as community transmission sources.

## Introduction

Norovirus (NoV, family *Caliciviridae*) causes nearly half of all acute gastroenteritis cases worldwide [1]. Norovirus infects people of all ages; however, the majority of reported data come from pediatric populations or outbreaks in adults from high-income countries. In adults, NoV infections have been well described in travelers [2] and persons working [3] or residing in hospitals [4], military camps [5], aboard ships [6, 7], and in other closed environments [1]. However, conclusions from these studies may not be generalizable to at-risk adults from low- and middle-income countries (LMIC), where NoV infections occur at a young age, and reinfection occurs frequently thereafter [8].

Noroviruses can be classified into six different phylogenetic clades, or genogroups (GI-GVI). Viruses from GI, GII and, much less frequently, GIV cause human disease. Norovirus genogroups are further classified into genotypes, and genotype GII.4 is further classified into variants. The emergence of novel GII.4 variants has initiated all six NoV-associated acute gastroenteritis pandemics noted since 1995 [9].

Given recent advances in the development of NoV candidate vaccines, there is a need to estimate the burden of NoV–associated disease in populations at risk for acute gastroenteritis worldwide, as well as to determine the predominant NoV genotypes and their associated pathogenicity [10–13]. However, there is limited information about the burden of NoV-associated adult gastroenteritis in LMIC settings. Therefore, we conducted a nested case-control study within a cohort of Peruvian military personnel in the Amazon Basin to characterize the burden of NoV infection, predominant genotypes, and associated symptoms in adults followed longitudinally in an LMIC setting.

## Materials and Methods

### Parent cohort study

**Fig 1:**
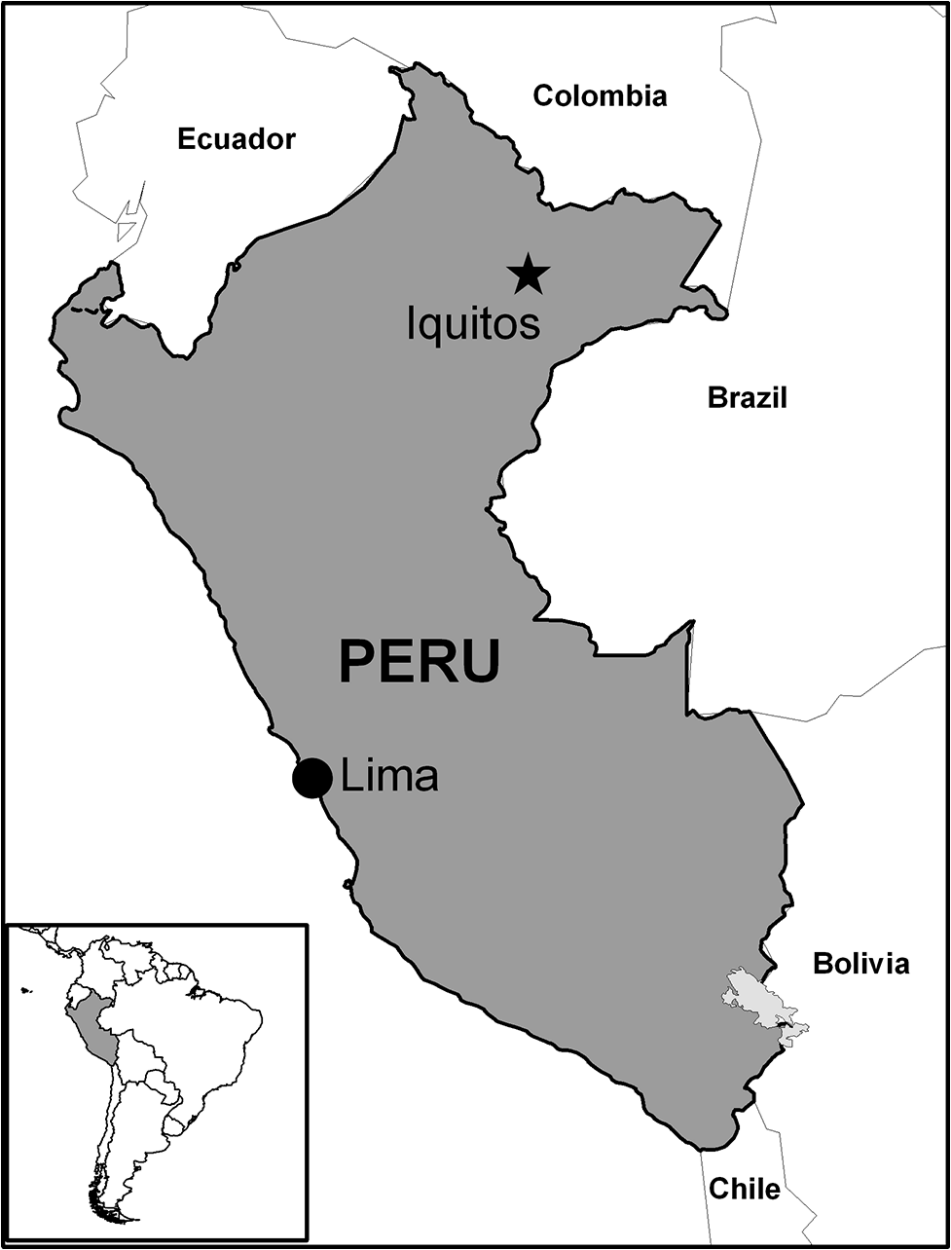
Map of Peru indicating the study site of Iquitos in the Peruvian Amazon Basin and the capital Lima, site of the main U.S. Medical Research Unit No. 6 laboratory.

Between January 1, 2002, and December 31, 2011, the United States Naval Medical Research Unit No. 6 (NAMRU-6) conducted surveillance for acute diarrhea among military personnel at the Vargas Guerra Army base, Iquitos, Peru (Fig 1). Diarrhea was defined as three or more loose or watery stools or two loose or watery stools accompanied by nausea, vomiting, abdominal cramps/pain, or tenesmus within the preceding 24 hours. After at least seven days without diarrhea following enrollment in the cohort study, asymptomatic participants provided basic demographic information and a stool sample (“baseline stool”). Passive surveillance (i.e. recording of persons presenting to the health center) for diarrhea was conducted until February 1, 2004, after which a study nurse conducted active surveillance, screening each participant daily for diarrhea. If a participant met the case definition, the study nurse recorded the patient’s oral temperature, administered a symptom questionnaire, and collected a fresh stool sample. At the conclusion of each diarrhea episode, the study nurse questioned the participant about the episode’s impact on daily activities. Impact was classified as mild if, as a result of the episode, the participant experienced no change in daily plans; moderate if the participant experienced an alteration in daily activities but did not miss work; or severe if the participant missed at least part of the work day. Two milliliters of fresh stool were separated for storage at -80°C for future NoV testing and the remainder sent to the NAMRU-6 laboratory in Iquitos for bacteriological and parasitological evaluation as described below.

### Nested case-control study

Within the described cohort, we conducted a nested case-control study using stool samples and data collected from participants who enrolled in the study during the active surveillance period from February 1, 2004, through December 31, 2011. Cases were participants with a first episode of diarrhea, as defined above. Controls were participants who were asymptomatic at the time of stool collection. We randomly selected 200 cases and 200 controls for inclusion in the nested case-control study.

### Norovirus detection and sequencing

A suspension of each stored stool specimen (10% wt/vol) was prepared in phosphate buffered saline and RNA extracted using the Qiagen QIAmp viral RNA kit (Valencia, CA) in accordance with the manufacturer’s instructions. Viral RNA was tested for NoV GI and GII by real-time polymerase chain reaction (PCR) [14]. A sample was considered positive if the negative control did not exhibit fluorescent curves. The threshold cycle for the sample was at least 37 for NoV GI and 39 for GII. Positive samples were then genotyped by sequencing Region C within the capsid gene after conventional PCR and gel-purification of amplicons, comparing results with NoV prototypic strains using the NoroNet sequence typing tool [15].

### Bacteria and parasite detection

At the time of collection, fresh stool specimens were transported in Cary-Blair medium and wide-mouthed containers to the NAMRU-6 laboratory in Iquitos for bacteria and parasite analysis, respectively. *Escherichia coli* and *Salmonella*, *Shigella*, *Yersinia*, and *Campylobacter spp*. were cultured and isolated as previously described [16, 17]. Polymerase chain reaction for heat labile and stable enterotoxigenic *E*. *coli* (ETEC) was performed on five lactose-fermenting colonies morphologically resembling *E*. *coli* [18]. *Plesiomonas spp*. were identified using the method of Hugh [19]. *Vibrio cholerae* were isolated using thiosulfate citrate bile salts sucrose agar [20]. Microscopy for ova and parasites was performed on saline wet preparations to detect protozoa and helminth infections. The Ritchie method was used to microscopically identify *Giardia spp* [21].

### Statistical methods

The 2-tailed Student *t* test was used for comparison of continuous outcome variable means. Proportions were compared using Fisher’s exact test. The relationship between dichotomous clinical signs and symptoms of gastroenteritis and predictor variables was assessed using univariate logistic regression. A sensitivity analysis was performed by re-classifying the NoV-bacterial co-infections as NoV negative and repeating the multivariate logistic regression analysis of the relationship between NoV infection and diarrheal disease. Norovirus pathogenicity indices (ratio of the prevalence of NoV in diarrheal stools/prevalence in baseline) were calculated.

The statistical analysis for predictors of diarrhea was performed in several steps; first, the association of diarrhea with specific pathogens was evaluated using simple and multiple logistic regression analyses. Conditional mean imputation was used to handle missing exposure values. Variance inflation factors were all below zero. Forward and backward stepwise selection was used to select variables for regression models. Nested models were compared using likelihood ratio tests and Akaike’s Information Criteria. When two nested models were compared using likelihood ratio tests, only the cases common to both regression models following model-wise deletion were used for comparison. Potential interactions were evaluated with regression models. We used adjusted odds ratios and pathogen prevalence among cases to calculate adjusted population attributable fractions (AFs) to estimate the pathogen-specific disease burden [22]. The adjusted AF is derived from the logistic regression model that includes other pathogens significantly associated with diarrhea; thus, it is the AF adjusted for the presence of other pathogens. Diarrhea was considered to be attributable to the pathogen identified in stools collected at the time of a diarrhea episode.

For diarrhea incidence calculations in the nested study, the average time to first diarrhea episode was calculated for the randomly selected cases, and average time of observation since enrollment or last diarrhea episode, whichever came first, was calculated for the randomly selected controls. To estimate total person-time observed during the period of active surveillance, these numbers were then multiplied by the number of cases and controls, respectively, enrolled during this period. The incidence of diarrhea was then calculated by dividing the number of cases by the total person-time. To calculate NoV diarrhea incidence, the prevalence of NoV in stools from cases was multiplied by the diarrhea incidence. Two-sided p-values less than 0.05 were considered statistically significant. STATA version 12.1 (College Station, Texas) was employed for all analyses.

### Ethics statement

This study was approved by the Naval Medical Research Unit No. 6 Institutional Review Board and the military command at Vargas Guerra Army base. Participants provided written informed consent.

## Results

### Parent cohort study

**Fig 2:**
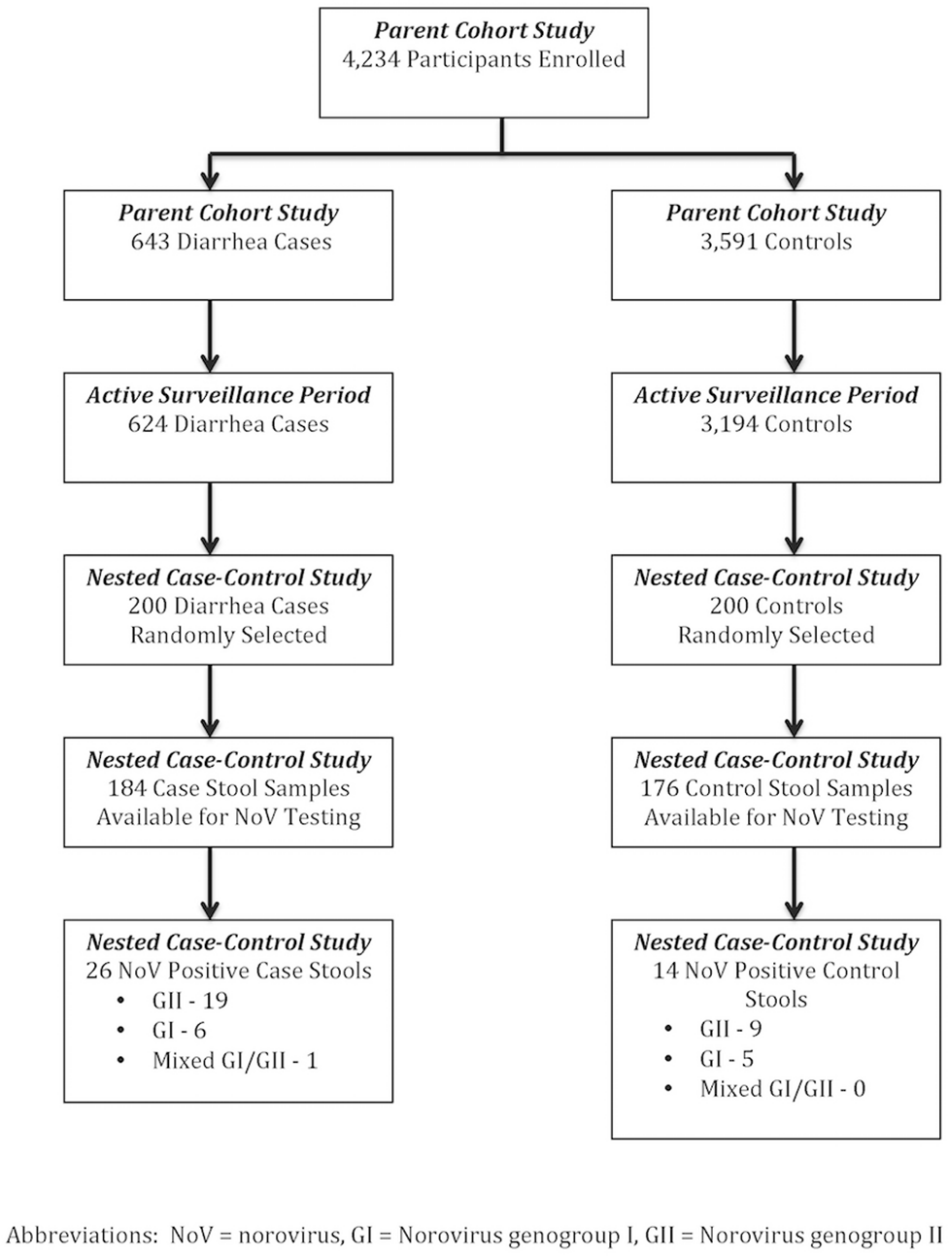
Flow diagram of participant enrollment, diarrhea outcomes, and selection for nested case-control study. Abbreviations: NoV = norovirus; GI = Norovirus genogroup I; GII = Norovirus genogroup II.

During the 10-year period of mixed active and passive surveillance, 4,234 males aged 18–34 were enrolled in the parent cohort study (Fig 2). Of these, 643 met the diarrhea case definition.

### Nested case-control study

During the 8-year active surveillance period, 3,194 participants were enrolled in the cohort, and 624 developed diarrhea (attack rate 19.5%). The incidence of diarrhea was 42.3 cases per 100 person-years. The 200 randomly selected diarrhea cases and 200 randomly selected controls were roughly evenly distributed over the study period (data not shown). Of these, 184 case and 176 control stools were available for NoV analysis. Norovirus was identified in 14.1% (26/184) and 8.0% (14/176) of stool samples from cases and controls, respectively (Table 1). The NoV-associated diarrhea attack rate was 2.7% (14.1% prevalence of NoV in case stools * 19.5% diarrhea attack rate) with an incidence of 6.0 NoV diarrhea cases per 100 person-years. The NoV pathogenicity indices were 1.4 for GI, 2.1 for GII, and 1.8 overall.

**Table 1:** Stool analysis results of cases verus controls, no. (%).

Organisms	Cases	Controls	*P*-Value
**Viruses**	(n = 184)	(n = 176)	
**Norovirus** *	26 (14.1)	14 (8.0)	0.067
**GI**	7 (3.8)	5 (2.8)	0.771
**GII**	20 (10.9)	9 (5.1)	0.053
**Bacteria**	(n = 200)	(n = 198)	
***Shigella***	**43 (21.5)**	**8 (4.0)**	**<0.001**
**ETEC †**	**16 (8.1)**	**2 (1.0)**	**0.001**
***Campylobacter***	1 (0.5)	4 (2.0)	0.214
***Salmonella***	3 (1.5)	0 (0.0)	0.248
***Plesiomonas***	2 (1.0)	1 (0.0)	>0.999
***Vibrio***	1 (0.5)	0 (0.0)	>0.999
***Yersinia***	0 (0.0)	0 (0.0)	>0.999
**Parasites**	(n = 196)	(n = 194)	
***Trichuris***	**30 (15.3)**	**52 (26.8)**	**0.006**
***Ascaris***	88 (45.4)	71 (36.2)	0.080
***Giardia***	42 (21.4)	38 (20.0)	0.707
***Uncinaria***	27 (13.5)	30 (20.1)	0.669
***Ancylostoma***	7 (3.6)	3 (1.6)	0.337
***Strongyloides***	2 (1.0)	7 (3.6)	0.104

Abbreviations: GI = norovirus genogroup I, GII = norovirus genogroup II, No. = number, ETEC = Enterotoxigenic *Escherichia coli*

*One participant with diarrhea tested positive for both norovirus GI and GII infection.

†RT-PCR for Enterotoxigenic *Escherichia coli* was not performed on 6 samples.

Diarrhea cases had a higher prevalence of ETEC (*P* = 0.001) and *Shigella* spp. (*P*<0.001) relative to controls, but a lower prevalence of *Trichuris* (*P* = 0.006) (Table 1, S1 Dataset). Multiple logistic regression analysis revealed NoV infection to be significantly associated with diarrhea (AOR 2.8, 95% CI: 1.3–6.0, *P* = 0.008). However, when stratified by genogroup, this relationship only remained significant for NoV GII (*P* = 0.012). The NoV GI variable was subsequently dropped from the adjusted model (Table 2). Other variables associated with diarrhea in the adjusted NoV GII model were infection with *Shigella* spp. (*P*<0.001), ETEC (*P* = 0.004), and *Trichuris* (*P* = 0.005), the latter being associated with a decreased risk of diarrhea. After reclassifying NoV-bacteria co-infected individuals as NoV-negative in the sensitivity analysis, the relationship between GII infection and diarrhea remained statistically significant in the adjusted multiple logistic regression model (AOR 2.7, CI: 1.0–7.1, *P* = 0.047). The AFs for NoV GII, *Shigella* spp., and ETEC were 6.4% (95% CI: 1.0–9.0%), 18.2% (95% CI: 14.3–20.0%), and 7.1% (95% CI: 3.8–7.9%), respectively.

**Table 2:** Results of logistic regression analysis of pathogen with diarrhea as the outcome variable.

Risk Factor	Crude			Adjusted^a^		
	OR	95% CI	*P*-value	OR	95% CI	*P*-value
NoV GII	2.4	(1.1–5.6)	0.03	3.4	(1.3–8.7)	0.012
*Shigella*	6.5	(3.0–14.2)	<0.001	6.7	(3.0–14.8)	<0.001
Enterotoxigenic *Escherichia coli*	8.5	(1.9–37.6)	0.005	9.1	(2.0–41.2)	0.004
*Trichuris*	0.5	(0.3–0.8)	0.006	0.4	(0.3–0.8)	0.005

Abbreviations: GI = Norovirus genogroup I, GII = Norovirus genogroup II, OR = Odds Ratio, CI = Confidence Interval

^a^ Adjusted model includes Norovirus GII, *Shigella*, Enterotoxigenic *Escherichia coli*, and *Trichuris*

Of all NoVs identified, 30.0% (12/40) were GI, 72.5% (29/40) were GII, and 2.5% (1/40) were mixed GI/GII infections. Twenty-five (61.0%) of the 41 NoV isolates could be genotyped (18 from cases and 7 from controls), of which 11 were NoV GI and 14 were GII (Table 3, S2 Dataset). The predominant NoV GI and GII genotypes were GI.4 (7 stools) and GII.4 (6 stools).

**Table 3:** Norovirus genotypes/varients detected in diarrhea caes versus controls, no., (%) ^a^.

Genotype/Variant (N = 25)	Total	Cases	Controls
**Genogroup I**			
**GI.1**	1 (4)	1 (4)	0 (0)
**GI.3**	1 (4)	1 (4)	0 (0)
**GI.4**	7 (28)	4 (16)	3 (12)
**GI.5**	1 (4)	0 (0)	1 (4)
**GI.7**	1 (4)	1 (4)	0 (0)
**Genogroup II**			
**GII.4/2006b Den Haag**	6 (24)	6 (24)	0 (0)
**GII.5**	1 (4)	1 (4)	0 (0)
**GII.6**	1 (4)	1 (4)	0 (0)
**GII.14**	1 (4)	1 (4)	0 (0)
**GII.15**	1 (4)	1 (4)	0 (0)
**GII.16**	2 (8)	2 (8)	0 (0)
**GII.17**	2 (8)	2 (8)	0 (0)
**Total**	25 (100)	21 (84)	4 (16)

Abbreviations: No. = number

^**a**^ In addition to those reported in the table, 16 NoVs were untypeable because no amplicon was produced on conventional PCR (0 NoV GI and 7 GII) or because there was no match for the sequence in GenBank (1 NoV GI and 8 GII).

Among all NoV-infected participants, co-infection with NoV and one or more organisms was present in 67.5% (27/40), including 12.5% (5/40) of participants infected with NoV and three other organisms. Norovirus co-infection was most often with chronically carried soil-transmitted helminths (n = 9), *Giardia* (n = 5), or both (n = 2), although co-infection with NoV and either *Shigella* (n = 4) or ETEC (n = 4) was also noted (Table 4). *Trichuris* was less frequently found in cases (15.3%, 30/196) relative to controls (16.8%, 52/194) (*P* = 0.006). However, among diarrhea cases, *Trichuris* was more frequent in GII-positive stools (30.0%, 6/20) relative to GII-negative ones (12.4%, 20/161) (*P* = 0.046). No other statistically significant associations were noted.

**Table 4:** Laboratory stool results for norovirus positive versus negative diarrhea cases, no. (%).

	Diarrhea Cases		
	Norovirus Negative(n = 158)	Norovirus Positive(n = 26)	*P*-Value
**Bacterial Pathogens**	(n = 158)	(n = 26)	
***Shigella***	36 (22.8)	4 (15.4)	0.608
**Enterotoxigenic *Escherichia coli***	12 (7.7)	4 (15.4)	0.253
***Salmonella***	3 (1.9)	0 (0.0)	>0.999
***Plesiomonas***	2 (1.3)	0 (0.0)	>0.999
***Campylobacter***	1 (0.6)	0 (0.0)	>0.999
***Vibrio***	1 (0.6)	0 (0.0)	>0.999
***Yersinia***	0 (0.0)	0 (0.0)	>0.999
**Parasites**	(n = 155)	(n = 26)	
***Trichuris***	19 (12.3)	7 (26.9)	0.067
***Ascaris***	57 (36.8)	6 (23.1)	0.191
***Giardia***	33 (21.3)	7 (26.9)	0.609
***Uncinaria***	23 (14.8)	2 (7.7)	0.539
***Ancylostoma***	6 (3.9)	0 (0.0)	0.596
***Strongyloides***	2 (1.3)	0 (0.0)	>0.999

Abbreviations: No. = number

^a^ Number of missing results were as follows: *Shigella*-0; ETEC-2; *Salmonella*-0; *Plesiomonas*-0; *Campylobacter*-0; *Vibrio*-0; *Yersinia*-0; *Trichuris*-3; *Ascaris*-3; *Giardia*-3; *Uncinaria*-0; *Ancylostoma*-3; *Strongyloides*-3.

Of NoV positive cases, 23.5% (6/26) reported that their symptoms had minimal impact on their daily activities, 73.1% (19/26) reported a moderate impact, and 3.8% (1/26) reported a severe impact. None of the participants were hospitalized and there were no deaths. There was no significant difference in the frequency of clinical symptoms associated with NoV diarrhea compared to other etiologies (data not shown). Infection with NoV GII produced more severe disease than NoV GI, reaching statistical significance for abdominal pain (94.7%, 18/19 versus 50.0%, 3/6, *P* = 0.031) (Table 5).

**Table 5:** Clinical features in diarrhea cases with GI versus GII norovirus, no. (%).

Clinical Feature	NoV GI(n = 6)^a^	Nov GII(n = 19) ^a^	*P*-Value
**Abdominal pain**	**3 (50.0)**	**18 (94.7)**	**0.031**
**Tenesmus**	3 (50.0)	14 (73.6)	0.344
**Abdominal cramps**	3 (50.0)	13 (68.4)	0.630
**Nausea**	1 (16.7)	5 (26.3)	>0.999
**Vomiting**	0 (0.0)	3 (15.8)	0.554
**Hematochezia**	0 (0.0)	1 (5.3)	>0.999
**Fever**	0 (0.0)	0 (0.0)	>0.999

Abbreviations: No. = number, GI = Norovirus genogroup I, GII = Norovirus genogroup II, No. = number, SD = standard deviation

^a^ The one participant co-infected with Norovirus GI and GII was excluded from this analysis.

## Discussion

Norovirus infection was the second most common diarrheagenic pathogen detected in our study. The virus, especially NoV GI, was also frequently present in stools of persons who had not recently experienced diarrhea, as has been frequently reported [23]. Furthermore, co-infection with NoV and other enteric organisms was frequent. In this pathogen diverse setting it can be difficult to determine the specific etiologic agent of a case of diarrhea. Nevertheless, the pathogenicity index of NoV, particularly the GII genogroup, was high relative to other major enteric pathogens. Norovirus was the sole pathogen in 27.0% of NoV-infected diarrhea cases and 69.2% of NoV diarrheal cases did not have a co-infecting bacterial pathogen. Together, these observations suggest that NoV is the cause of the majority of the diarrheal illness with which it is associated and a major cause of diarrhea in the study population. Possible reasons for the presence of NoV in baseline stools include continued shedding in persons recovering from a bout of diarrhea experienced before the wash-out period (NoV, particularly GII, can be shed for weeks to months after an episode of diarrhea)[2, 8], asymptomatic infection due to pre-existing innate or acquired immunity, and insufficient infectious dose.

Few studies have described the disease burden of NoV in adults in LMIC settings. The burden of acute diarrhea attributable to NoV noted in our study is similar to that noted in children in Peru [8, 24], although the risk of infection in our closed population on a military base is likely higher than that typically noted in adults in the community. Interestingly, in a rural community-based study of NoV by Peñataro Yori *et al* in children in the same region as our study site, the proportion of diarrheal cases attributable to NoV was highly age dependent, accounting for 22–25% in children 0–2 years old but steadily falling with age, accounting for less than 5% by age 4–5 years [24]. The authors concluded that naturally acquired immunity protected children as they age. However, our results suggest that many adults remain susceptible to infection and disease from NoV. We cannot determine whether the cases of NoV diarrhea in our study were a result of immune evasion by viral evolution or were a result of first infection in naïve hosts, although given that NoV infection in children is nearly universal in Peru [8], we highly suspect the former. The specific NoV genotypes circulating at the time of each study may also play a role, as immunity is likely genotype-specific [1]. Norovirus genotypes were not determined in the Peñataro Yori *et al* study. Another possible factor that could contribute to variable susceptibility of adults to NoV-induced diarrhea, not determined in either study, is histo-blood antigen group, which appears to mediate NoV-binding through epithelial cell surface ligands [25]. It is also worth noting that Yori *et al* reported fever in 71.4% of children with NoV-associated diarrhea versus none of the adults in our study, suggesting that at least some clinical features of NoV infection may be age dependent, or perhaps change with successive NoV infections.

A successful NoV vaccine will need to provide protection against various NoV genogroups and types [26]. Our investigation provides detailed data on the NoV genotypes circulating in the study region. As with many other studies, we found NoV GII to be a prevalent and particularly pathogenic genotype (especially GII.4), suggesting that it should be at least one major target of a NoV vaccine. Similar findings have been reported in Brazil and Argentina [27–29]. Previous research has shown that, compared with persons infected with other NoV genotypes, those infected with NoV GII.4 shed the virus in larger numbers [30], transmit the virus at higher rates [31], and have poorer outcomes [32]. Further investigation of the NoVs that we were unable to genotype will be important to fully understand the epidemiology and the organisms and epitopes relevant to a successful vaccine.

The range of organisms and co-infections noted in our study is similar to that previously reported in both children and adults in the region [24, 33]. Interestingly, co-infection with *Trichuris* was associated with a decreased risk of diarrhea in persons also infected with NoV. *Trichuris* co-infection in children has been documented with numerous enteric organisms, including *Entamoeba histolytica* [34], *Shigella* spp. [35], *Salmonella* spp. [35], and a variety of helminths [36]. We speculate that *Trichuris* infection in our population serves as a proxy for frequent previous infection and consequent enhanced immunity to enteric pathogens, including NoVs. The apparent lack of a protective effect of *Trichuris* infection against NoV GII infection relative to GI infection may relate to the increased mutation rate and thus genetic variability of the NoV GII genogroup, allowing these viruses to present varied epitopes that escape immune system detection [9].

Limitations to our study include the 1) Lack of a validated diarrhea severity score for adults, which limited our ability to evaluate the relationship between NoV infection and disease severity; 2) Use of a case definition that did not include vomiting without diarrhea, which is common with NoV infection, which could result in underestimation of the contribution of NoV to gastroenteritis in the study population; 3) Short (1-week) washout period before baseline stool evaluation, which could result in underestimation of the NoV pathogenicity index, given that NoV may be shed for a median of 28 days in adults following acute infection [37]; 4) Use of qualitative rather than quantitative PCR, precluding exploration of possible associations between viral load and disease; 5) Use of conventional PCR, which is less sensitive than rRT-PCR, to obtain material for genetic sequencing, which could have biased detection toward NoV genotypes and variants present in higher viral loads (Table 3). Nor would our methodology detect NoV GIV; 6) Lack of data on the precise duration of each participant on the base, which may have led to overestimation of the person-time at risk and an underestimation of incidence rates; and 7) Small sample size, which often limited statistical analysis of the temporal distribution and disease associations of specific NoV genotypes and variants. This latter point is especially important considering that NoV pandemics featuring novel variants occur every two to four years [9]. Rapid NoV mutation and recombination events may result in the development of novel NoV genotypes and variants, particularly in the GII genogroup, in a manner similar to that of the influenza A virus [38]. In settings where NoV is prevalent, mutations may occur at an accelerated pace, leading to the emergence of new variants capable of causing community epidemics.

In conclusion, our study helps extend knowledge of the complex epidemiology of adult NoV infection and disease in a closed setting in a LMIC. Such populations, which are traditionally prone to NoV outbreaks with high transmission rates, could serve as potential sources of introduction into the community and thus must be considered in future control and vaccination strategies.

## Supporting Information

S1 Dataset (XLS)

S2 Dataset (XLSX)
